# The C-Reactive Protein/Albumin Ratio as a Predictor of Mortality in Critically Ill Patients

**DOI:** 10.3390/jcm7100333

**Published:** 2018-10-08

**Authors:** Ji Eun Park, Kyung Soo Chung, Joo Han Song, Song Yee Kim, Eun Young Kim, Ji Ye Jung, Young Ae Kang, Moo Suk Park, Young Sam Kim, Joon Chang, Ah Young Leem

**Affiliations:** 1 Department of Pulmonary and Critical Care Medicine, Ajou University School of Medicine, Suwon 16499, Korea; jedkdl@yuhs.ac; 2 Department of Medicine, Yonsei University College of Medicine, Seoul 03722, Korea; 3 Division of Pulmonology, Department of Internal Medicine, Institute of Chest Disease, Severance Hospital, Yonsei University College of Medicine, Seoul 03722, Korea; chungks@yuhs.ac (K.S.C.); augustin76md@yuhs.ac (J.H.S.); dobie@yuhs.ac (S.Y.K.); narae97@yuhs.ac (E.Y.K.); stopyes@yuhs.ac (J.Y.J.); mdkang@yuhs.ac (Y.A.K.); pms70@yuhs.ac (M.S.P.); chang@yuhs.ac (J.C.)

**Keywords:** C-reactive protein, albumin, intensive care unit, mortality

## Abstract

The C-reactive protein (CRP)/albumin ratio has recently emerged as a marker for poor prognosis or mortality across various patient groups. This study aimed to identify the association between CRP/albumin ratio and 28-day mortality and predict the accuracy of CRP/albumin ratio for 28-day mortality in medical intensive care unit (ICU) patients. This was a retrospective cohort study of 875 patients. We evaluated the prognostic value of CRP/albumin ratio to predict mortality at 28 days after ICU admission, using Cox proportional hazard model and Kaplan-Meier survival analysis. The 28-day mortality was 28.0%. In the univariate analysis, the Acute Physiology and Chronic Health Evaluation II (APACHE II) score (*p* < 0.001), CRP level (*p* = 0.045), albumin level (*p* < 0.001), and CRP/albumin ratio (*p* = 0.032) were related to 28-day mortality. The area under the receiver operating characteristic (ROC) curve (the area under the ROC curves (AUC)) of CRP/albumin ratio was higher than that of CRP for mortality (0.594 vs. 0.567, *p* < 0.001). The cut-off point for CRP/albumin ratio for mortality was 34.3. On Cox proportional-hazard regression analysis, APACHE II score (hazards ratio (HR) = 1.05, 95% confidence interval (CI) = 1.04–1.07, *p* < 0.001) and CRP/albumin ratio (HR = 1.68, 95% CI = 1.27–2.21, *p* < 0.001 for high CRP/albumin ratio) were independent predictors of 28-day mortality. Higher CRP/albumin ratio was associated with increased mortality in critically ill patients.

## 1. Introduction

It is important to predict the prognosis of patients who require critical care in the intensive care unit (ICU), to determine the direction of treatment. To date, many studies have evaluated parameters or biomarkers used for the prediction of prognosis in critically ill patients. However, these factors are difficult to determine, and the immediate application of such prognostic information is complicated when dealing with unstable patients. A simple, quick, and accessible parameter is needed to confirm treatment response and predict mortality in ICU patients.

C-reactive protein (CRP) is an acute-phase protein that is produced following stimulation by various cytokines in response to infection, ischemia, trauma, and other inflammatory conditions [1]. High CRP levels have been studied in relation to prognosis and mortality in critically ill patients [2,3,4,5,6]. On the other hand, low serum albumin is known to be associated with poor prognosis and mortality [7,8]. Based on this knowledge, we speculated that the ratio of CRP to albumin could be used as a predictive marker for mortality.

Recently, the CRP/albumin ratio, a combination of markers for systemic inflammation and nutritional status, has been extensively studied as an independent prognostic marker in patients with infection, malignancy, and other diseases [9,10,11,12,13,14,15]. However, there are relatively few studies conducted focusing on critical care patients in the ICU [10].

In this study, we aimed to evaluate the association between the CRP/albumin ratio and prognosis in critically ill patients in the ICU.

## 2. Materials and Methods

### 2.1. Patients and Study Design

This retrospective study was conducted at the Severance Hospital of Yonsei University, a tertiary medical institution. Patients admitted to the medical ICU from 1 March 2015 to 30 September 2017 were included. The medical ICU is a 30-bed closed unit that is managed by certified ICU specialists. We excluded pediatric patients under 19 years and patients with chronic hepatitis or liver cirrhosis due to a possible effect on albumin levels.

For patients who were admitted to the ICU more than once during the study period, only the first admission data were recorded. Patients without major data or patients transferred from other ICUs were excluded.

### 2.2. Data Collection

Baseline characteristics; laboratory findings, including CRP and albumin concentrations; and disease severity, including the acute physiology and chronic health evaluation II (APACHE II) score, were included to evaluate the association with mortality in ICU patients. The laboratory tests were conducted within 24 h of ICU admission, and disease severity was assessed on ICU admission. The primary outcome was 28-day all-cause mortality.

### 2.3. Statistical Analysis

Categorical variables were presented as absolute frequency and percentage, and continuous variables were presented as mean ± standard deviation. The clinical characteristics of the two groups, survivors and non-survivors, were compared using the Pearson’s chi-squared test or Fisher’s exact test for categorical variables, and the Student’s *t*-test for continuous variables. For the logistic regression analysis, univariate analysis was performed first, and *p* < 0.05 was considered statistically significant [16,17]. Additionally, multivariate analysis was performed using the forward stepwise data selection method [16,17]. Cut-off values of 0.05 and 0.1 were applied to either enter or remove a covariate into or from the final model, and the results were described as odds ratio (OR) and 95% confidence intervals (CIs). The Cox proportional hazard regression analysis was used to evaluate the association between variables and mortality. The effect of age, sex, body mass index (BMI), underlying diseases, nutrition status, and APACHE II score was evaluated using Cox proportional hazard models, with an adjusted *p*-value < 0.05 considered statistically significant. Hazard ratios (HRs) and 95% CIs were calculated. Furthermore, survival was estimated using the Kaplan-Meier method, and differences in survival between groups were assessed using the log-rank test. Analysis of the receiver operating characteristic (ROC) curves and the area under the ROC curves (AUC) were performed to evaluate the CRP/albumin ratio as predictive values for mortality in critically ill patients. In addition, we compared the ROC curves between the CRP and CRP/albumin ratio. The primary outcome analysis was conducted via Kaplan-Meier analysis. A *p*-values < 0.05 were considered statistically significant. All statistical analyses were performed with IBM SPSS software version 23.0 (IBM, Armonk, NY, USA) and MedCalc Statistical Software version 12.7.7 (MedCalc Software bvba, Ostend, Belgium).

### 2.4. Ethics

The protocol was approved by Severance Hospital institutional review board (IRB No. 4-2018-0094). The need for informed consent was waived given the retrospective nature of the study.

## 3. Results

**Figure 1:**
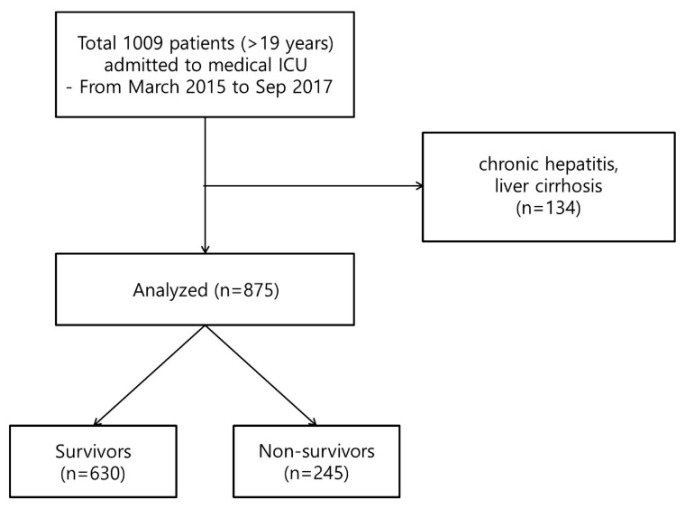
Flowchart of the inclusion and exclusion process for enrollment of patients in the study. ICU: intensive care unit. A total of 1009 patients were enrolled, and 875 patients were included in the analysis.

A total of 1009 patients older than 19 years were admitted to the medical ICU, excluding duplicate admissions of patients who were re-admitted during the study period. We excluded patients with chronic hepatitis or liver cirrhosis, resulting in a total of 875 patients included in the study (Figure 1). The mean age was 66.4 ± 14.7 years. The most common underlying disease was hypertension (*n* = 485, 55.4%), followed by diabetes mellitus (*n* = 260, 29.7%); and malignancy was found in 229 (26.2%) of these patients. The mean APACHE II scores were 24.5 ± 9.1, respectively. The 28-day mortality was 28.0% (*n* = 245); these patients were defined as non-survivors. Remaining patients were defined as survivors. The comparison of demographic, clinical, and laboratory parameters between non-survivors and survivors is shown in Table 1. In univariate analysis, age (*p* = 0.014) and APACHE II score (*p* < 0.001) were significantly higher in non-survivors. Non-survivors had a higher prevalence of underlying cancer (*p* < 0.001), acute renal failure (*p* < 0.001), and sepsis (*p* = 0.031). Furthermore, in laboratory findings, serum hematocrit (*p* = 0.004), platelet count (*p* < 0.001), and albumin level (*p* < 0.001) were lower in non-survivors than in survivors. CRP level (*p* = 0.045) and CRP/albumin ratio (*p* = 0.032) were significantly higher in non-survivors than in survivors (Table 1).

In the multivariate analysis, adjusted for age, sex, BMI, underlying disease, and APACHE II score, APACHE II score (odds ratio (OR) = 1.06, 95% confidence interval (CI) = 1.042–1.079, *p* < 0.001), history of cancer (OR = 1.595, 95% CI = 1.04–2.30, *p* = 0.012), and CRP/albumin ratio (OR = 1.01, 95% CI = 1.00–1.02, *p* = 0.001) were independent predictors of 28-day mortality (Table 2). Additionally, subgroup analysis was performed except for patients with solid cancer or hematologic malignancy who are currently undergoing treatment or whose cancer is seen in the imaging evaluation during the study period. In the multivariate analysis, APACHE II score (odds ratio (OR) = 1.08, 95% confidence interval (CI) = 1.062–1.105, *p* < 0.001), and CRP/albumin ratio (OR = 1.01, 95% CI = 1.002–1.009, *p* = 0.002) were independent predictors of 28-day mortality.

**Figure 2:**
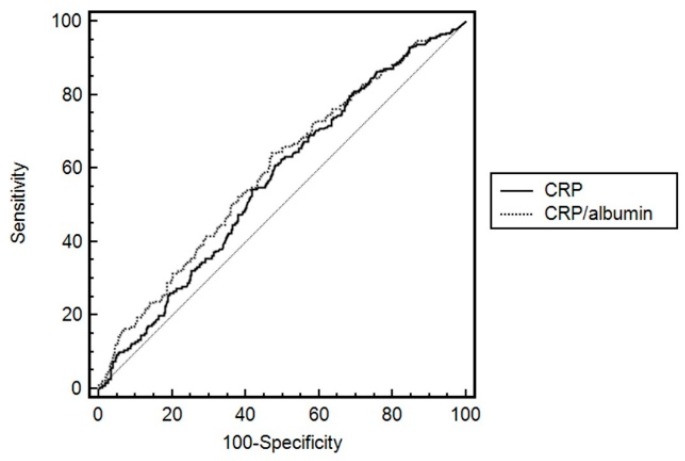
ROC curves of CRP and CRP/albumin ratio as 28-day mortality predictors. The AUC of CRP/albumin ratio was higher than that of CRP for mortality in ICU patients (*p* < 0.001). ROC, receiver operating characteristic; CRP, C-reactive protein; AUC, area under the curve.

Figure 2 shows the ROC curves of CRP and CRP/albumin ratio. After performing a ROC analysis and calculating the AUC, the AUC of CRP/albumin ratio was higher than that of CRP for mortality in ICU patients (0.594 vs. 0.567, *p* < 0.001). The optimal cut-off point of CRP/albumin ratio was 34.3 for 28-day mortality after medical ICU admission, and the sensitivity and specificity for 28-day mortality were 64.2% and 52.7%. Based on these results, all patients were classified into the high-CRP/albumin ratio (CRP/albumin ratio > 34.3) and low-CRP/albumin ratio (CRP/albumin ratio ≤ 34.3) groups. The ROC analysis in patients without malignancy disease was performed additionally. Even though cancer patients were excluded, the AUC of CRP/albumin ratio was higher than that of CRP for mortality in critically ill patients (0.585 vs. 0.559, *p* < 0.001).

**Figure 3:**
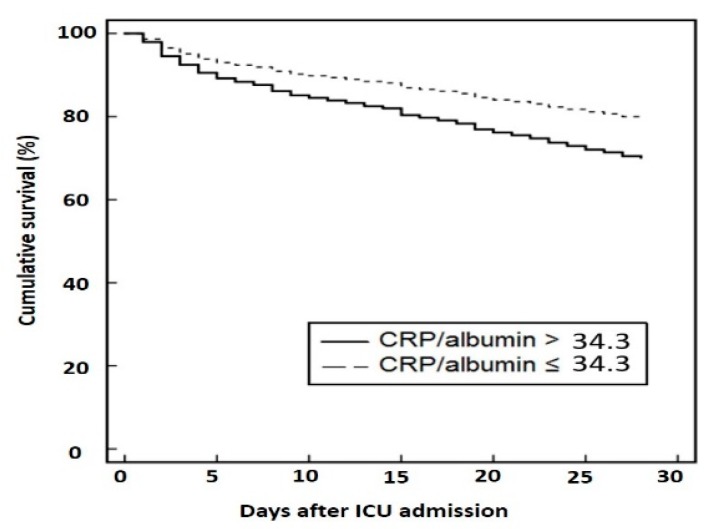
Kaplan-Meier survival curve according to the serum CRP/albumin ratio. For survival analysis, patients were divided into two groups according to CRP/albumin ratios. The cut-off value was 34.3. The relative risk of mortality was significantly associated with the CRP/albumin ratio (*p* = 0.001).

The results of the survival analysis are shown in Figure 3 and Table 3. In the Kaplan-Meier survival analysis, a higher CRP/albumin ratio (>34.3) was significantly related to higher 28-day mortality rates (*p* < 0.001) (Figure 3). The effects of age, sex, BMI, APACHE II score, underlying disease, and CRP/albumin ratio on 28-day mortality were analyzed using Cox proportional hazard model (Table 3). For survival analysis, patients were divided into two groups according to CRP/albumin ratios. The relative risk for mortality was significantly associated with APACHE II score (hazards ratio (HR) = 1.05, 95% CI = 1.04–1.07, *p* < 0.001) and CRP/albumin ratio (HR = 1.68, 95% CI = 1.27–2.21, *p* < 0.001 for high CRP/albumin ratio). In subgroup analysis for patients without malignancy disease, the relative risk for mortality was also associated with APACHE II score (hazard ratio (HR) = 1.04, 95% CI = 1.03–1.06, *p* < 0.001) and CRP/albumin ratio (HR = 1.39, 95% CI = 1.02–1.90, *p* = 0.04 for high CRP/albumin ratio).

## 4. Discussion

In the current study, we found that the CRP/albumin ratio determined from data collected on admission was significantly related to higher 28-day mortality in critically ill patients in the medical ICU. Based on the ROC curve, cut-off values of 34.3 for the CRP/albumin ratio were found to be predictive of 28-day mortality. The relevance of these results are strengthened by the large number of patients included in this study. These results add to the current body of knowledge concerning the role of the CRP/albumin ratio as a prognostic factor for mortality in critically ill patients.

CRP levels have been shown to be associated with various conditions, including severe sepsis, heart failure, cerebral disease, and other inflammatory diseases [2,3,4,18,19,20,21]. Furthermore, CRP has been used as a prognostic marker in the critical care setting [2,3,4]. In previous studies, CRP concentration at ICU discharge was an independent predictor for higher risk of readmission [3] and in-hospital mortality after ICU discharge [2]. In a study by Villacorta et al. CRP was an independent cardiovascular mortality predictor in patients with acute decompensated heart failure [4]. However, in other studies, CRP levels alone did not successfully predict survival outcomes in patients with sepsis [22,23]. Silvestre et al. found no correlation between CRP levels and sepsis severity, organ failure, and ICU mortality [22]. In their, CRP levels poorly predict mortality outcomes [22]. In a study of a mixed medical-surgical intensive care unit, serum CRP concentration measured on the day of discharge from intensive care was not a predictor of readmission or death [23]. In ICU in non-immune-compromised septic patients, monocyte-human glucocorticoid receptor (hGR) protein and extracellular heat shock protein-72 (eHSP72) were strong predictors of mortality (area under receiver operating characteristic (AUROC) > 0.95, *p*  <  0.04) [24]. Resistin and extracellular heat shock protein-90α (eHSP90α) (AUROC > 0.85) could better than CRP or lactate (AUROC > 0.75) discriminate sepsis from systemic inflammatory response syndrome (SIRS) (*p* < 0.001) in adults [25]. Therefore, the use of CRP for prognosis in critically ill patients remains controversial.

On the other hand, hypoalbuminemia is common in seriously ill patients, and serum albumin level has been associated with increased mortality in acutely ill patients in previous reports [7,26]. In a meta-analysis of 90 cohort studies, hypoalbuminemia was a dose-dependent predictor of poor outcomes, such as mortality, morbidity, and prolonged ICU and hospital stay [26]. The association between hypoalbuminemia and poor clinical outcomes appeared to be independent of both nutritional status and inflammation in that study [26]. In a single-site prospective cohort study carried out in a medical-surgical ICU of a tertiary care center, APACHE II score and albumin level were independent risk factors for mortality in ICU patients with severe sepsis or septic shock [7]. As a potential outcome predictor, serum albumin level has been added as a component parameter in the APACHE III score [27]. However, hypoalbuminemia can be caused by previous illness or general conditions, such as liver disease, kidney damage, and malnutrition [28,29,30]. Therefore, it is difficult to use CRP or albumin as a biomarker alone [31].

In combination, CRP and albumin may be more valuable prognostic markers for outcomes across various diseases, providing both inflammatory and nutritional information [32,33]. The combination of albumin and CRP into a single index has been suggested previously, and subsequent studies have shown that the CRP/albumin ratio is more consistent with prognosis than CRP or albumin alone [10]. The CRP/albumin ratio has been extensively studied as an independent prognostic marker in patients with infection, malignancy, and other diseases [9,10]. Kim et al. reported that the CRP/albumin ratio at admission was positively correlated with prognosis in patients with severe sepsis or septic shock treated with early goal directed therapy [9]. In that study, the cut-off value for the CRP/albumin ratio as predictor of mortality was 5.09 in patients with severe sepsis or septic shock [9]. In a study of elderly patients admitted via the emergency room, high-sensitivity-CRP/albumin ratio at admission to the emergency department was associated with all-cause in-hospital mortality among patients older than 65 years [32]. The CRP/albumin ratio has been shown as a predictor of mortality in acute pancreatitis patients [34]. Furthermore, the CRP/albumin ratio has predicted overall survival in various malignancies [11,12,13,14,15]. On the other hand, there have been relatively few studies conducted focusing on critical care patients in the ICU. Ranzani et al. reported that the CRP/albumin ratio was an independent predictor of 90-day mortality in 334 ICU patients with severe sepsis or septic shock, using a CRP/albumin ratio cut-off value of 8.7 at ICU admission and 2.0 at discharge [10]. Similarly, a modified nutritional Index (NI) including CRP and the more sensitive than albumin transthyretin (CRP × Fibrinogen/Transferrin × Transthyretin), along with CRP and Interleukin (IL)-10, was shown to increase in pediatric ICU non-survivors [35]. In our study, the model using CRP/albumin ratio had greater accuracy than CRP alone for predicting mortality. Furthermore, the CRP/albumin ratio was an independent risk factor for mortality at 28 days in critically ill patients and is consistent with previous studies. This study differed from other previous studies as it included a mixed caseload of ICU patients, not solely patients with sepsis or malignancy. The effect of the inflammatory response associated with critical illness, other than cancer and infection, may affect these results.

This study has several strengths and limitations. While there are many scoring systems used to predict the prognosis of critically ill patients, the CRP/albumin ratio is valuable because it is relatively simple and easy to use in all settings. However, there are several limitations of this study. A limitation that may have biased results is the inclusion of cancer patients in the analysis of the critically ill. It has been previously shown that high pretreatment CRP/albumin ratio indicates poor prognosis in human malignancies in the study reported by Hong-jun Xu et al. [36]. However, in our subgroup analysis excluding cancer patients, the CRP/albumin ratio was also an independent predictor of 28-day mortality in the multivariate analysis. Additionly, the relative risk for mortality was significantly associated with APACHE II score and high CRP/albumin ratio in Cox proportional hazard model. Second, Immuno-compromised patients such as HIV infection, solid organ transplant and autoimmune disease were included. These patients with chronic diseases can affect serum CRP and albumin levels. While, the number of patients is not significant, it is considered to be a limitation of this study. Third, the sensitivity and specificity for 28-day mortality were not good for prediction. However, the CRP/albumin ratio had more accuracy than CRP alone for predicting mortality. The CRP/albumin ratio is significant useful because it is available easily in general institutions. Since these strengths, it may be meaningful in spite of limitations. Fourth, this observational study was a single-center, retrospective cohort study, making the generalization of findings difficult. However, the sample size was relatively large, considering that this was a study conducted at only one institution. Fifth, because the serum concentrations of CRP and albumin were only analyzed at ICU admission, we could not evaluate the changes in CRP, albumin, and CRP/albumin ratio over time. Spanaki et al. reported that CRP decreases longitudinally among ICU survivors whereas SIRS survivors showed a longitudinal decline in albumin [37]. Further studies that investigate the changes in these markers are needed in the future.

In conclusion, this study showed that a higher CRP/albumin ratio is associated with increased mortality in ICU patients. However, the sensitivity and specificity of CRP/albumin ratio for prediction of mortality were not so high in this single center study. Therefore, large, multicenter studies are required to confirm these results and establish the serum CRP/albumin ratio as a useful marker for the prediction of prognosis in critically ill ICU patients.

## Figures and Tables

**Table 1:** Baseline characteristics of patients at day 0 of ICU admission *.

Variables	Non-Survivors	Survivors	*p*-Value
	(*n* = 245)	(*n* = 630)	
Age, years of age	68.2 ± 13.4	65.5 ± 15.0	0.014
Male (%)	311 (61.6)	137 (60.6)	0.805
BMI, kg/m^2^	22.1 ± 4.7	22.0 ± 4.9	0.725
Disease severity			
APACHE II score	29.2 ± 9.5	22.7 ± 8.3	<0.001
Underlying diseases			
Diabetes mellitus	67 (27.3)	193 (30.6)	0.339
Chronic lung disease ^†^	36 (14.7)	113 (17.9)	0.252
Hypertension	134 (54.7)	351 (55.7)	0.785
Heart failure	30 (12.2)	81 (12.9)	0.807
Coronary artery disease	41 (16.7)	83 (13.2)	0.175
Cancer	88 (35.9)	141 (22.4)	<0.001
Acute renal failure	97 (39.6)	166 (26.3)	<0.001
ARDS	32 (13.1)	60 (9.5)	0.126
Sepsis	182 (75.1)	426 (67.6)	0.031
Laboratory parameters			
WBC (10^3^/µL)	13.8 ± 11.8	14.7 ± 9.5	0.675
Hct (%)	28.2 ± 7.3	29.7 ± 6.5	0.004
Platelets, 10^3^/mm^3^	125.6 ± 105.5	180.4 ± 121.8	<0.001
Albumin, g/dL	2.4 ± 0.6	2.6 ± 0.5	<0.001
Creatinine, mg/dL	2.1 ± 1.9	2.7 ± 15.0	0.579
Procalcitonin, ng/mL	1.8 ± 2.2	1.5 ± 2.0	0.781
CRP, mg/L	125.2 ± 108.1	111.1 ± 96.7	0.045
CRP/Albumin ratio	50.9 ± 54.3	43.9 ± 46.9	0.032

ICU, intensive care unit; BMI, body mass index; APACHE II, Acute Physiology and Chronic Health Evaluation II; ARDS, acute respiratory distress syndrome; WBC, white blood cell; Hct, hematocrit; CRP, C-reactive protein. * Data are presented as number (percentage) or mean ± standard deviation, unless otherwise indicated. ^†^ Chronic lung disease includes asthma, chronic obstructive pulmonary disease, and structural lung diseases, such as bronchiectasis and interstitial lung disease.

**Table 2:** Multivariate analysis to predict 28-day mortality after ICU admission.

Variables	OR	95% CI	*p*-Value
Age	1.00	0.99–1.02	0.601
Sex, F/M (%)			
Male	Reference	Reference	Reference
Female	1.18	0.83–1.66	0.358
BMI	0.99	0.96–1.01	0.254
APACHE II score	1.06	1.04–1.08	<0.001
Underlying diseases			
Cancer	1.59	1.11–2.30	0.012
CRP/Albumin	1.01	1.00–1.02	0.001

ICU, intensive care unit; OR, odds ratio; CI, confidence interval; F/M, male/female; BMI, body mass index; APACHE II, Acute Physiology and Chronic Health Evaluation II; CRP, C-reactive protein.

**Table 3:** Cox proportional hazard regression analysis for 28-day mortality.

Variables	HR	95% CI	*p*-Value
Age	1.00	0.99–1.01	0.992
Sex			
Male	Reference	Reference	Reference
Female	1.23	0.93–1.61	0.142
BMI			
APACHE II score	1.05	1.04–1.07	<0.001
Underlying diseases			
Cancer	1.30	0.98–1.71	0.071
CRP/Albumin			
Low (≤34.3)	Reference	Reference	Reference
High (>34.3)	1.68	1.27–2.21	<0.001

HR, hazards ratio; CI, confidence interval; BMI, body mass index; APACHE II, Acute Physiology and Chronic Health Evaluation II; CRP, C-reactive protein.
